# “*We were leery of going*”: qualitatively exploring Canadian international retirement migrants’ travel-related decisions during the COVID-19 pandemic

**DOI:** 10.1186/s40794-024-00218-z

**Published:** 2024-05-01

**Authors:** Jessica Tate, Valorie A. Crooks, Jeremy Snyder

**Affiliations:** 1 Department of Geography, Simon Fraser University, Burnaby, BC Canada; 2 Faculty of Health Sciences, Simon Fraser University, Burnaby, BC Canada

**Keywords:** International retirement migration, Canada, United States, Decision-making, COVID-19 pandemic

## Abstract

**Background:**

International retirement migration, which is the seasonal or permanent relocation of older people to another country, has grown in popularity in recent years. These retirees are motivated by the promise of warmer winter climates that are conducive to participating in health-promoting recreational and social activities. Ease of cross-border travel facilitates this transnational practice when undertaken seasonally. However, border closures and other travel-related measures put in place to manage the spread of COVID-19, disrupted travel, including for older Canadians who typically winter in the United States (US). During the 2020-21 winter season, for example, Canadians were advised not to engage in non-essential international travel and the land border between Canada and the US was closed to all but essential travellers. Nonetheless, retirement migration remained a significant draw for many Canadian retirees. Here, we qualitatively explore the factors that Canadian international retirement migrants considered when deciding whether or not to travel to the US for the 2020-21 winter during the COVID-19 pandemic.

**Methods:**

Guided by case study methodology, semi-structured interviews were conducted with 31 Canadian international retirement migrants who had wintered in the US prior to the COVID-19 pandemic and were in the US at the outset of the pandemic in late winter 2020. Interviews were transcribed verbatim and thematically analyzed to decipher what factors were most important to their travel-related decision-making during the pandemic. We structure the thematic results around four factors previously identified to motivate older people to become international retirement migrants and thus inform decision-making: the destination, the people, the cost, and the movement.

**Results:**

The previously identified factors that motivate older people to participate in international retirement migration include: the destination (e.g., climate and amenities), the people (e.g., social networks), the cost (e.g., health insurance and living costs), and the movement (e.g., ease of travel). These factors informed how international retirement migrants made decisions to travel abroad or not in the 2020-21 winter season. For example, destination-based factors included a lack of public health measures and high case counts, people-based factors comprised of less opportunities to engage in social activities, cost-based factors involved maintaining property investments and the lack of COVID-19 treatment coverage in available travel health insurance plans, and movement-based factors included challenges in ease of access when travel was viewed as essential or non-essential. These factors disincentivized or motivated international retirement migrants to travel abroad in the 2020-21 winter season during the COVID-19 pandemic.

**Conclusions:**

The results of this study support the need to create tailored decision-support tools for international retirement migrants to make informed travel-related decisions during crisis events so as to protect their health and wellbeing. More research is needed to explore perceptions of risk, especially health risks, among international retirement migrants and how they differently affect their travel-related decisions.

## Background

Some retirees travel abroad seasonally to enjoy warmer climates and their associated benefits for health and wellbeing, such as participating in outdoor recreational opportunities and creating social networks. This practice, known as international retirement migration, is growing in popularity [1]. A recent scoping review indicates that people are motivated to become international retirement migrants for four main reasons: the destination (e.g., health promoting opportunities); the people (e.g., opportunities to create new social networks); the cost (e.g., affordability of the destination); and the movement (e.g., travel options) [2]. These motivating factors inform decision-making regarding whether or not to participate in international retirement migration as well as destination selection. Two noteworthy factors that further support retirees participating in this practice are having the financial resources to allow travel while maintaining two residences (whether owned or rented) along with travellers’ personal health status [2–4]. The financial costs of wintering abroad are sometimes partially offset by lower costs of living in some destinations, though even in such cases new seasonal expenses may still be introduced such as those associated with purchasing travel health insurance policies and/or transportation [2].

Hundreds of thousands of older Canadians are among those retirees internationally who travel abroad for the winter, typically for stays of weeks or months between November and April [5]. The United States (US) is a popular destination for them due to its proximity and the ease of travelling there by vehicle [5]. The southern states of Florida, Texas, Arizona, and California are particularly popular among older Canadian retirement migrants [6–8]. Many residential communities in these states actively seek to attract Canadian retirees through offering appealing amenities such as seniors recreational complexes, hospitals and health care clinics with geriatric expertise, year-round security, and organized social activities [9–11].

Although Canadians enjoy access to publicly funded medically necessary health care at home, this coverage is not portable internationally, creating a barrier to international retirement migration [12]. Thus, many Canadian international retirement migrants purchase private travel health insurance in order to protect their health while wintering in the US [8, 13, 14]. Canadian international retirement migrants have reported that these policies can be costly, sometimes prohibitively so, and complex to navigate in terms of fully understanding care inclusions/exclusions [13]. The challenging complexity of these policies has also been acknowledged by administrators and clinicians working at hospitals in popular US destinations for older Canadians [8]. For example, navigating the Canadian travel medicine insurance policies has proven to be a significant challenge for international retirement migrants where they have reported having to wait for insurance approvals from Canada which many US health care professionals are unfamiliar with [8]. Other challenges to obtaining travel health insurance include language barriers, a lack of trust in health care systems abroad, along with migrants perceived need of health care while abroad [15].

The global COVID-19 pandemic, which was officially declared in March of 2020 by the World Health Organization, affected the travel and movements of intended international retirement migrants, including Canadians, for many reasons. Canadian international retirement migrants were among those who were requested to return home quickly at the outset of the pandemic in March, 2020 before border closures or other public health measures restricted their transnational movements [16, 17]. By March 21 the shared land border between the US and Canada had closed to all non-essential travel and by March 25 quarantine orders were put in place for Canadians returning from abroad, including returning retirement migrants [18, 19]. This border closure measure remained in effect until November, 2021 when the shared land border reopened and vaccinated Canadians could once again travel by vehicle to the US for recreational purposes [20]. The land border closure lasted a total of 19 months and spanned the entirety of the 2020-21 winter season when hundreds of thousands of older Canadians would typically have travelled to the US (primarily by land) for weeks or months as retirement migrants. It is this winter season that serves as the focus of our paper, where we explore what motivated experienced Canadian international retirement migrants to travel to the US, or not, during the height of the pandemic when non-essential travel was restricted.

Here we present a qualitative analysis exploring which factors influenced Canadian international retirement migrants in their decision-making about the possibility of travelling to the US for the 2020-21 winter season, which was at a point in the COVID-19 pandemic when Canadians were advised not to engage in non-essential travel due to personal and public health risks. As we point out in the discussion, understanding factors important to international retirement migrants’ travel decision-making during a global health crisis can be useful in developing tailored decision-support tools for this group of travellers that can be used in other crises affecting travel and movement and have personal and public health implications (e.g., environmental disasters). The development of such tools is critical because as Pickering et al. (2021) recently found, there is little information available that is tailored to Canadian international retirement migrants, in particular, to support them in making informed travel-related decisions that will ultimately protect their health and wellbeing [13].

## Methods

We used an exploratory case study methodological approach to examine the decision-making factors motivating Canadian international retirement migrants to winter in the US, or not, during the COVID-19 pandemic. We are a team of social science and humanities health researchers, two of whom have over a decade of experience qualitatively studying health-related transnational movements including international retirement migration (VC, JS), one of whom has parents who previously participated in international retirement migration (VC), and two of whom have no lived or familial experience of this practice (JT, JS). Case study methodology was selected as it allows for the comprehensive exploration of an idea or issue within a specific context which is undertaken through reviewing multiple sources of data. In addition to gathering the primary data reported on in this analysis, we also separately examined Canadian newspaper articles discussing the portrayal of international retirement migrants and their travel throughout the pandemic in mainstream Canadian print news media as part of the case study that is published elsewhere [17]. Given that international retirement migrants’ experiences during the COVID-19 pandemic have been influenced by a range of contextual factors, such as changing border regulations, case study provides the tools to understand these complex situations through a focus on context [21]. To get a deeper understanding of Canadian international retirement migrants’ travel-related decisions in the context of the COVID-19 pandemic, one-on-one interviews were conducted as they allow for capturing in-depth insights [22].

Participant recruitment started following approval of this study from our institution’s research ethics board. Given the exploratory nature of this study, we aimed to conduct at least 20 phone interviews with eligible Canadian international retirement migrants. We conducted the interviews over a two-month period (Dec /21-Jan/22), using a temporal cut-off to guide data collection. The inclusion criteria were: (1) having been in the US for the 2019/20 winter season (i.e., at the outset of the pandemic), (2) having gone to the US or not gone to the US for the 2020/21 winter season; and (3) being able to participate in an English-language phone interview. Multiple recruitment strategies were employed simultaneously. They included: (1) posting in popular Facebook groups for Canadian international retirement migrants, along with replying to social media threads on Twitter, Facebook, or news articles featuring issues salient to the participant group identified through keyword searching; (2) advertising the study on Kijiji and Craigslist, which are popular online classified advertisement websites used in Canada; (3) reaching out to known contacts from our previous studies with Canadian international retirement migrants who opted to be informed about further studies; (4) our own personal and professional networks; and (5) inviting existing participants to share study information with eligible others. Prospective participants who reached out were sent a follow-up email that included study details and information on the consent process. Those who were still inclined to participate were asked to reply to the first author by email to confirm eligibility and were asked to send potential interview dates and times. Upon confirmation of eligibility, interviews were scheduled based on participants’ availability.

The phone interviews were approximately 1 h in length, all of which were conducted by the first author to enhance consistency. All interviews were digitally recorded. A semi-structured interview script was used to guide the interviews that prompted discussion of experiences of returning to Canada from the US at the outset of the pandemic in early 2020 and probed decision-making regarding going abroad the following winter (2020-21), including sources of information to support decision-making. Specific interview prompts were informed in part by a scoping review of motivations for participating in international retirement migration that identifies decision-making factors [2], and by the newspaper article analysis noted above as being a component of this case study [17]. At the end of the interview, each participant was invited to discuss any relevant experiences that were not already discussed during the interview. After, interview participants received a CAD $25 e-gift card to a retailer of their choosing to acknowledge their valuable contributions to the study.

Interview recordings were transcribed verbatim using a paid transcription service upon completion. The first author reviewed transcripts for completeness and accuracy as they were delivered, augmenting any unclear sections by returning to the audio recording to add additional notes (this was done minimally as the audio recordings were sound and the transcripts were found to be of high quality). To proceed with analysis, each author independently reviewed five unique transcripts in preparation for analysis. Following an independent review, a team meeting was held to identify emergent themes and discuss any outliers in preparation for thematic analysis [23]. We reached consensus about three distinct emergent meta-themes central to the narratives participants shared, one of which centred around travel decision-making and is presented herein. We contrasted these three emergent meta-themes against the relevant existing literature, which is an important step in thematic analysis [24], to assist with defining the scope and scale of each. Our next step was to create a coding scheme to assist with organizing the data relevant to each meta-theme to identify sub-themes. A scheme was developed collaboratively and implemented by the first author with any uncertainties in interpretation being resolved by the second author. Coding was conducted manually by the first author, with excerpts relating to each code in the scheme being extracted from the transcripts and organized using a synthesis table shared via GoogleDocs. It was at this point we agreed that the four motivating factors identified in the Pickering et al. (2019) scoping review served as a meaningful framework for exploring the sub-themes connected to the travel decision-making meta-theme and so we integrated them into the synthesis table for the current analysis [2]. Upon completion of coding, we independently reviewed the synthesis tables to confirm the interpretation of the coded excerpts and the analytic scope. Overall, rigour was built into our analytic process through the use of investigator triangulation throughout that allowed for the confirmation of findings through differing perspectives [25, 26].

## Results

We interviewed 31 Canadian international retirement migrants, 11 women and 20 men, who were abroad in the 2019-20 winter season at the outset of the COVID-19 pandemic and who did or did not return to the US for the following winter season. With regard to the 2020-21 winter season, thirteen participants chose to winter in the US while the majority remained in Canada. Participants resided in three different Canadian provinces, and all were experienced retirement migrants who typically wintered in the states of Florida or Arizona with their marital partners. Eighteen participants owned seasonal properties in the states of Florida or Arizona, while 13 typically rented accommodations seasonally or drove to the US in their personal motorhomes.

As noted in the introduction, winter 2020-21 was a point in the COVID-19 pandemic at which Canadians were advised to avoid international travel, including to the US, unless it was considered essential. The Canada-US land border was specifically closed to non-essential travel. In the remainder of this section, we explore what motivated participants to either travel to the US or to stay at home in Canada for the 2020-21 winter season in light of these and other public health measures put in place at that time. We organize these motivations around the four factors that Pickering et al.’s (2019) scoping review found were most important in informing decision-making regarding participating in international retirement migration: the destination (e.g., climate and amenities), the people (e.g., social networks), the cost (e.g., health insurance and living costs), and the movement (e.g., ease of travel) [2]. In the sub-sections that follow we consider each of these factors in relation to participants’ motivations to travel during the COVID-19 pandemic. In each sub-section we contrast the perspectives of those who opted to go abroad for the 2020-21 winter against those who did not.

### The destination

Participants saw their usual retirement migrant destinations in two distinctly different ways during the 2020-21 winter. For those who opted to winter in the US, their usual destinations were viewed as safe and health-promoting places to spend time during the pandemic. This was generally consistent with how they understood these same winter destinations in pre-pandemic years and the potential they offered for engaging in what was considered a healthier lifestyle in a warmer climate than what they would experience at home in Canada. Wintering in the US provided an important perceived benefit for most who opted to travel. Being in a warmer climate was thought to have protected them from COVID-19 transmission risks as they were able to engage in outdoor socialization and recreation opportunities that were not available at home. For example, a participant who opted to go to the US explained the importance of this decision-making factor:

Well, do we go down south [to the US] and enjoy the warm weather and be outdoors in our own yard and just take care that we don’t go anywhere? Or do we stay in the apartment building [in Canada] with 71 other apartments where you’re walking common property where people are sneezing, and sniffing, and coughing, and touching things, and you’re indoors over the crappy winter? And we said, ‘no, we’re going south’.

A destination-based amenity that motivated some participants to travel during the pandemic was the potential for earlier access to COVID-19 vaccines while in the US than at home in Canada, with some reporting they were able to access their first vaccine “immediately” upon arrival in the US. Outside of this opportunity, participants who opted to travel generally viewed the usual destination-related motivations for engaging in international retirement migration including a warmer climate that offers health benefits as protective factors for wintering abroad during the pandemic.

For participants who did not go abroad in the 2020-21 winter, their typical retirement migrant destinations were commonly characterized as risky settings. Such assessments were often made by watching or reading local news reports while home in Canada and connecting with friends to learn about protective practices, such as mask-wearing, and the general local sentiment about the state of the pandemic. These concerns weighed heavily throughout decision-making regarding winter travel, as one participant explained:

We were leery of going down [to the US]. Even friends that we have that live in Florida permanently in the [residential] park, they were hesitant about going out and going anywhere. So, the more we heard from even the locals…it was, “Nope, we don’t want to go.” The other thing was, the park, while they did implement a number of [COVID-19 related] restrictions, they were certainly a lot less than what we would’ve seen up here [in Canada].

Many participants who were motivated to stay at home for the winter closely followed COVID-19 case counts in their usual US destinations, with a participant noting that “Canada wasn’t the best, but we had better numbers and it was safer to be home. And the people here, I think, were more responsible. So that was the big thing.” Another common concern among participants who opted not to travel was the perception that pandemic-related public health measures, such as mask-wearing mandates and business closures, were inadequate in their US destination. Overall, destination-based factors such as the absence of public health measures, high case counts, word-of-mouth from friends, and local news coverage of the pandemic served as both disincentives from travelling and motivators to stay in Canada.

### The people

Participants who opted to travel to the US for the 2020-21 winter were commonly drawn to do so to maintain their usual winter social networks. Though they recognized that fewer Canadian international retirement migrants would likely be in the destination, it was thought that there would be greater opportunities for socialization abroad than at home. Some opted to travel abroad for the winter because their usual destination had fewer pandemic-related measures or restrictions in place that would inhibit social interactions relative to their home communities in Canada. For example, one participant explained:

There were only two of us that came down. All the rest of the Canadians stayed home. But once we arrived there, it was like, oh my god, a whole new world opened up to us because everything was open. People wore masks down there, but it was none of this 50% capacity for restaurants… I mean, at our stage in life to be locked down, you’re not living, you’re surviving.

Overall, even in their more limited form, the potential for socialization in public and community settings, such as recreation centres and restaurants, was a significant decision-making factor for those who opted to travel to the US for the 2020-21 winter when pandemic-related travel restrictions were in place in Canada.

Participants who opted to stay in Canada for the 2020-21 winter generally acknowledged that they were sacrificing the social lives and networks they enjoyed while normally wintering in the US. One participant remarked that “in one way, we have good friends down there [in the normal US destination]. We enjoyed it down there. But the bottom line is things have changed. COVID has changed.” Concern was not raised by these same participants, however, that social relations with their usual wintertime friends could not be resumed once travel restrictions were eased and retirement migration was possible again. Thus, the loss of social interactions for the 2020-21 winter was viewed as temporary, as opposed to permanent, and many participants reported looking forward to resuming friendships in their usual wintertime destinations once non-essential travel was allowed again.

### The cost

For participants who owned properties in the US, a significant factor in their decision-making about whether or not to go abroad in the 2020-21 winter was the need for household maintenance. For some, their winter homes were seen as an investment that could not be abandoned or disregarded, even in a time of crisis. A participant explained that their seasonal home, “it’s just not something that you oddly leave.” This perspective drove some participants to make the decision to go abroad for the winter. An important financial facilitator for travel was the ability to access affordable travel health insurance that covered COVID-19-related treatment. As one participant explained, “my husband has a policy…and they do insure for COVID, they did allow COVID insurance. We’re pretty healthy, we don’t really have any pre-existing conditions, so we felt we were pretty safe coming down then.” Participants who opted to travel generally also identified economic incentives for wintering abroad during the pandemic, such as the lower cost of food and goods in the US that could offset higher travel health insurance policy costs.

While some participants were able to afford the high cost of travel health insurance during the COVID-19 pandemic, others found this insurance inaccessible. Having private travel health insurance is not a requirement for Canadians travelling abroad. However, participants who felt these policies were important to hold consistently remarked about the costs of such insurance during the pandemic or the fact that policies were not available to them at all. For example, one participant explained that they were unable to purchase policies because they had contracted COVID-19 too close to when they wanted to depart for the US. Another remarked that the idea of not being adequately protected against the potentially high costs of being treated for COVID-19 while in the US “scared us”, even if they were able to afford a travel health insurance policy. Such possibilities weighed heavily in these participants’ minds when deciding whether or not to travel to the US for the 2020-21 winter.

### The Movement

Although all participants were aware of the Canada-US land border closure and request for Canadians to avoid non-essential travel during the 2020-21 winter, some suggested there were “inconsistencies” in how these measures were enforced. These inconsistencies enabled some participants to circumvent restrictions through creativity, such as by flying to their US destination while also shipping their personal vehicle or recreational vehicle through a freight transportation provider across the land border. For example, one participant explained that “when the land border closed and we decided to ignore the recommendation that came from the government, we ended up flying that year… I think as long as we could get a flight to go to our home in Phoenix [Arizona] and we felt very safe.” Another suggested inconsistency came in relation to interpretations of what constituted non-essential travel. Participants who opted to travel to the US for winter 2020-21 consistently viewed their travel as being essential due to factors such as owning a property in the destination that needed to be maintained or the personal health benefits they experienced from being in a warmer climate for the winter months. Therefore, understanding one’s own seasonal travel to be ‘essential’ was an important decision-making factor considered by these participants.

The participants who did not opt to travel abroad collectively agreed it was their responsibility to follow the Canadian government’s request to avoid non-essential international travel and viewed their wintertime stays abroad as non-essential. By avoiding their usual transnational movements during the peak of the pandemic, they were doing what they could to protect their health and support the general safety and wellbeing of others. Participants who opted not to travel to the US reported thinking very carefully about their decisions, often weighing multiple factors. Even if some factors supported decisions to travel, “in the end, we just decided, no, it wasn’t safe” or was not essential for the participant, all things considered. It was also acknowledged that there were exceptional costs related to travel during the pandemic, such as shipping personal vehicles or purchasing COVID-19 tests that were required upon re-entry into Canada, which served as an additional barrier to transnational movement.

## Discussion

Every winter tens of thousands of Canadian retirees temporarily relocate to international destinations, typically having been motivated to do so by destination-, cost-, people-, and movement-related factors [2]. The COVID-19 pandemic disrupted this transnational movement as a result of land border closures and preventive public health measures that had a profound impact on older Canadians’ seasonal travel-related decisions [17]. Among these impacts were the sometimes-challenging decisions Canadian international retirement migrants needed to make about whether or not to winter in the US during the pandemic. Our interviews with 31 of these older Canadians revealed a range of factors they took into consideration when making decisions about the possibility of engaging in seasonal travel, which are synthesized in Table 1. This synthesis table contrasts the findings of this analysis against the usual motivating factors for participation in international retirement migration previously identified by Pickering and colleagues [2]. As shown in Table 1, the four broad motivations identified by Pickering and colleagues were salient to older Canadians’ decision-making about wintering in the US during the pandemic, but they informed decisions both to travel and also to stay at home among participants [2]. Different factors also emerged to shape travel-related decision-making during the pandemic relevant to each of these broad motivations. For example, participants spoke little about the general cost of living (e.g., less expensive groceries) as motivating their travel to the US during the pandemic while instead focusing greatly on the need to travel to maintain costly investment properties despite public health advisories to avoid doing so.

**Table 1:** Synthesis of travel-related decision-making factors considered by Canadian international retirement migrants during the COVID-19 pandemic

	How this factor usually motivates people to participate in international retirement migration (as per Pickering et al., 2019)	How this factor motivated Canadians to participate in international retirement migration during the COVID-19 pandemic	How this factor deterred Canadians from participating in international retirement migration during the COVID-19 pandemic
*The Destination*	Having access to a preferred climate that brings health benefits and associated amenities	Having access to a preferred climate that brings health benefits and associated amenities	Local news reporting framing the destination as risky; High COVID-19 case counts; Perceiving a lack of preventative public health protocols in the destination
*The People*	Developing new social networks; The potential for participating in new cultural practices; Spending time with people from home while abroad	Maintaining established social networks in the destination; Greater opportunities to socialize safely in a warmer climate	Protecting personal health and those of other Canadians by avoiding travel abroad during the closure of the land border and requests to avoid non-essential international travel
*The Cost*	Affordable cost of living, including accommodations and health care, in the destination	Maintaining property investments in the destination	Inaccessibility of affordable travel health insurance that included COVID-19 coverage
*The Movement*	Ease of travel and few or no regulatory barriers to long-stay visits	Viewing travel as essential despite land border closure and requests to avoid non-essential international travel	Viewing the closure of the land border and requests to avoid non-essential international travel as absolute barriers to travel

All participants in this study shared the experience of having been in the US at the outset of the COVID-19 pandemic at the end of the 2019-20 winter season, often having to navigate unexpected returns home to Canada. Yet, despite this common experience, the findings shared above show that participants had very different understandings of the risks associated with traveling to the US for the 2020-21 winter. For example, while some who were unable to access affordable travel health insurance covering COVID-19 infections viewed this as an absolute barrier to travel, others felt comfortable staying in the US for the winter season without any such coverage. This finding echoes Pickering and colleagues’ recent qualitative analysis of the strategies that Canadian international retirement migrants use to manage their health while abroad [13]. That study found that while it was not uncommon for these older travellers to opt not to purchase travel health insurance policies, in some cases they would make the decision to do so in an uninformed or haphazard manner without having conducted adequate research or consulted with experts, such as their own physicians [13].

Another important difference that emerged among participants pertained to their views of whether or not they considered their winter travel to be essential. One of the strongest justifications for viewing their travel as essential during the pandemic was home ownership in the destination community. Gibler and colleagues created a logistic regression model that explored home preferences among international retirement migrants [27]. The affordability and practicality of home maintenance is an important enabling factor in their model. They contend that once retirement migrants are not able to maintain their homes properly due to health, financial, or other reasons, they are more likely to consider selling and/or moving. Interview participants who justified their travel to be essential due to home ownership - despite the Canadian government’s request not to engage in non-essential international travel during winter 2020-21 - may have been grappling with an awareness that if they were unable to keep up with routine maintenance due to a lack of access this may negatively affect their abilities to continue as dual property owners (i.e., at home and abroad) in the future. Such acknowledgement may have supported some homeowning participants’ justification of the decision to take the risk of international travel during a global public health crisis.

The onset of the COVID-19 pandemic was unsettling for many Canadian international retirement migrants who were abroad at its outset due to stressful journeys back to Canada along with quickly needing to navigate their newly disrupted lives [17, 28]. This same group had to then make important travel-related decisions regarding whether or not to go abroad the following winter (i.e., winter 2020-21) based on insights from their personal networks and other information that was available to them, which was described by participants as “inconsistent”. This analysis shows that Canadian international retirement migrants did not commonly have access to reliable third-party information to support informed decision-making about travel during the pandemic. Others have found similar informational gaps, like Hatz and colleagues, who contend that global travel-related disruptions caused by public health measures put in place during the COVID-19 pandemic were done on an unprecedented scale and there was a general unpreparedness with regard to providing practical information for international travellers [29]. It was evident that participants were impacted by this lack of preparedness and had to, quite independently, identify sources of information that they deemed reliable, to support their decision-making about wintering in the US during the pandemic. Suh and Flaherty (2019) argue that older travellers greatly benefit from having access to tailored information that can support their health while travelling, which is what participants lacked [30]. Meanwhile, Pickering and colleagues contend that such tailored information to support the health-related travel decisions of Canadian international retirements generally does not exist [13]. Given the potential for future pandemics, environmental disasters, and/or other crises to cause similar travel disruptions, it would be useful for public health officials in retirement migrants’ home and destination communities to prepare and/or assemble relevant decision-support information. Such an informational tool can meaningfully cover details related to: travel health insurance; property access and maintenance; interpreting travel advisories; local resources to ensure health and wellbeing; and details on reliable sources of information.

This analysis has a number of strengths, including capturing nuanced details of Canadian international retirement migrants’ travel decision-making that add new insights to the existing literature, giving voice to these participants through the inclusion of verbatim quotes in the findings section, and our use of investigator triangulation to enhance the rigour of data analysis. There are, however, limitations. A significant one is that we only conducted interviews and English and did not provide opportunities for French-speaking Canadian international retirement migrants, nor those who do not speak either of Canada’s official languages, to participate. This limitation was due to language competency among our investigative team. Capturing the lived experiences of linguistically diverse Canadian international retirement migrants is certainly a direction for future research on decision-making among this group. Another limitation is that the lack of population-level data on Canadian international retirement migrants, including the US destinations they visit and length of stay, means that it is not possible to characterize how reflective those with whom we spoke are of this group as a whole. Although such statistical or population generalizability is not a goal of qualitative research, it would still be helpful to have a sense as to how this participant group is situated in relation to other older Canadians who winter in the US.

The findings of this analysis hold a number of implications for further research, three of which we highlight. First, it has been suggested that the mobility disruptions and impacts of the COVID-19 pandemic hold parallels to other types of crisis events at the local or regional level, such as environmental disasters [31, 32]. As such, there is great potential to learn from international retirement migrants’ experiences of the COVID-19 pandemic so as to identify transferable points that can assist with better supporting this group in other crisis events. Such research has the potential to inform the types of informational tools we call for above. Second, Pickering et al. (2019) identified ease of travel as a dominant motivation for participating in international retirement migration and that much research on this practice is focused on travel between countries with few entry or exit requirements [2]. The current analysis has shown that when ease of travel is disrupted there are significant consequences for international retirement migrants. Outside of our focus on pandemic-related disruption, considerable discussion is emerging in the policy community about how British retirees who winter in European Union countries (e.g., Spain, France) are likely to encounter new types of travel disruptions as a result of changed border access brought on by Brexit [33, 34]. Future research should deeply explore the types of expectations and assumptions retirees hold about continuing ease of access to destinations when incorporating international retirement migration into their plans so as to begin to identify opportunities for supporting realistic planning. Finally, this analysis identified that obtaining affordable travel health insurance can be an important determining factor to enable or prevent Canadian international retirement migrants from travelling abroad, at least in a global public health crisis situation. The role that travel health insurance plays in supporting (or deterring) international retirement migration tends to be mentioned peripherally in existing research [2], and so future research should more meaningfully explore the complex relationship between insurance providers and retirement migrants in the travel decision-making process.

## Conclusions

This analysis has aimed to qualitatively explore international retirement migration by Canadians to the US during the COVID-19 pandemic, specifically related to travel-related decisions for the 2020-21 winter season. Through thematic analysis of 31 interviews, we identified a number of factors that were important to such decisions (see Table 1). Factors that supported decisions to go abroad despite travel advisories to the contrary included the desire to maintain social networks, engage in outdoor recreation, and maintain owned properties abroad. Factors that supported decisions to stay in Canada included a desire to follow public health messaging and the lack of affordable travel health insurance packages that included COVID-19 coverage. The differing ways in which such factors were considered and the resulting decisions highlight arguments in recent studies that point to the fact that international retirement migrants are not a homogeneous group, despite often being characterized as such [13, 35].

Overall, the findings of this analysis show that the COVID-19 pandemic disrupted the practice of international retirement migration by creating challenges associated with travelling amid a global health crisis. These challenges included disrupted land border access, struggles in obtaining affordable and/or adequate travel health insurance, and most significantly, the absence of third-party information needed to support informed travel-related decision-making by Canadian international retirement migrants. We thus call for the development of decision-support tools tailored to the informational needs of international retirement migrants to use in other global or local crises that similarly impact both health and mobility so as to support this group in making informed travel-related decisions.

## Data Availability

To maintain participants’ privacy and anonymity, interview transcripts from this study are not publicly available.

## Abbreviations

US: United States
